# Mycobacterial chaperonins in cellular proteostasis: Evidence for chaperone function of Cpn60.1 and Cpn60.2‐mediated protein folding

**DOI:** 10.1111/mmi.15109

**Published:** 2023-06-23

**Authors:** Bakul Piplani, C. M. Santosh Kumar, Peter A. Lund, Tapan K. Chaudhuri

**Affiliations:** ^1^ Kusuma School of Biological Sciences Indian Institute of Technology Delhi India; ^2^ School of Biosciences University of Birmingham Birmingham; ^3^ Institute of Microbiology and Infection University of Birmingham Birmingham UK

**Keywords:** chaperonin 60, GroEL, molecular chaperones, *Mycobacterium tuberculosis*, protein folding, proteostasis

## Abstract

*Mycobacterium tuberculosis* encodes two chaperonin proteins, MtbCpn60.1 and MtbCpn60.2, that share substantial sequence similarity with the *Escherichia coli* chaperonin, GroEL. However, unlike GroEL, MtbCpn60.1 and MtbCpn60.2 purify as lower‐order oligomers. Previous studies have shown that MtbCpn60.2 can functionally replace GroEL in *E. coli*, while the function of MtbCpn60.1 remained an enigma. Here, we demonstrate the molecular chaperone function of MtbCpn60.1 and MtbCpn60.2, by probing their ability to assist the folding of obligate chaperonin clients, DapA, FtsE and MetK, in an *E. coli* strain depleted of endogenous GroEL. We show that both MtbCpn60.1 and MtbCpn60.2 support cell survival and cell division by assisting the folding of DapA and FtsE, but only MtbCpn60.2 completely rescues GroEL‐depleted *E. coli* cells. We also show that, unlike MtbCpn60.2, MtbCpn60.1 has limited ability to support cell growth and proliferation and assist the folding of MetK. Our findings suggest that the client pools of GroEL and MtbCpn60.2 overlap substantially, while MtbCpn60.1 folds only a small subset of GroEL clients. We conclude that the differences between MtbCpn60.1 and MtbCpn60.2 may be a consequence of their intrinsic sequence features, which affect their thermostability, efficiency, clientomes and modes of action.

## Graphical abstract

*Mycobacterium tuberculosis* encodes two enigmatic GroEL homologs, MtbCpn60.1 and MtbCpn60.2. Here, we study their functions by examining their effects on cellular phenotypes associated with obligate chaperonin clients. We show that MtbCpn60.1 supports cell survival and division to a limited extent by assisting the folding of a small subset of obligate chaperonin clients, and MtbCpn60.2 efficiently supports cell growth and proliferation by assisting the folding of a larger client pool. This is the first report demonstrating that both MtbCpn60.1 and MtbCpn60.2 are bona fide molecular chaperones.

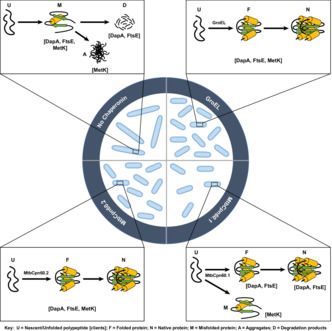

**Abbreviations**
Cpn10: 10 kDa co‐chaperonin
Cpn60: 60 kDa chaperonin
DAP: Meso‐2,6‐diaminopimelic acid
DapA: 4‐hydroxy‐tetrahydrodipicolinate synthase
FtsE: cell division ATP‐binding protein FtsE
MetK: S‐adenosylmethionine synthase
MtbCpn10: *M. tuberculosis* 10 kDa co‐chaperonin
MtbCpn60.1: *M. tuberculosis* 60 kDa chaperonin 1
MtbCpn60.2: *M. tuberculosis* 60 kDa chaperonin 2

## 1 INTRODUCTION

Molecular chaperones are a diverse superfamily of proteins that are crucial for maintaining cellular proteostasis. Chaperonins are a family of ubiquitous, ATP‐dependent molecular chaperones known for their characteristic multimeric ring‐shaped assembly (Horwich & Fenton, 2020; Saibil & Ranson, 2002). In the bacterial cytosol, chaperonins typically exist as ‘cages’ consisting of two heptameric rings stacked back‐to‐back that are capped by dome‐shaped heptamers of the co‐chaperonin (Braig et al., 1994; Hayer‐Hartl et al., 2016; Saibil, 1996; Sigler et al., 1998; Xu et al., 1997; Yan et al., 2018). Each ring of the chaperonin cage has a large central cavity to encapsulate unfolded client proteins, sequestering them in an isolated environment that favours productive folding unperturbed by cytosolic macromolecular crowding (Balchin et al., 2016; Ellis, 2003; Hartl & Hayer‐Hartl, 2002; Horovitz et al., 2022). Each chaperonin monomer has a three‐domain architecture—a client and co‐chaperonin binding apical domain, an equatorial domain responsible for ATPase activity and inter‐subunit interactions, and a bridging intermediate domain that facilitates inter‐domain communication (Braig et al., 1994; Horwich & Fenton, 2020; Saibil et al., 2013). Chaperonin and co‐chaperonin monomers have molecular weights of approximately 60 and 10 kDa, respectively, and hence, the proteins are often designated as Cpn60 and Cpn10 (Coates et al., 1993).

Current understanding of chaperonin and co‐chaperonin properties is largely derived from research on the *Escherichia coli* GroEL and GroES proteins, respectively. GroEL and GroES assist the folding of about 10–15% of the *E. coli* proteome, including several essential proteins, and hence are essential for cell viability (Chapman et al., 2006; Ewalt et al., 1997; Fayet et al., 1989; Fujiwara et al., 2010; Houry et al., 1999; Kerner et al., 2005). Bacterial chaperonins are highly conserved proteins, therefore, *E. coli* GroEL has been regarded as a good model for all bacterial chaperonins. However, while *E. coli* has a single *groES‐groEL* operon, about 30% of the bacterial species with completely sequenced genomes have multiple *cpn60* genes (Kumar et al., 2015; Lund, 2009) which may exhibit functional divergence (Goyal et al., 2006; Mande et al., 2013). This phenomenon was first reported in *Mycobacteria* (de Wit Rinke et al., 1992) that have two (rarely three) *cpn60* genes but only one *cpn10* gene (Colaco & Macdougall, 2014; Lund, 2009). The deadly pathogen *Mycobacterium tuberculosis* has two chaperonin genes, herein referred to as *Mtbcpn60.1* and *Mtbcpn60.2* (Kong et al., 1993; Lund, 2009). While *Mtbcpn60.2* is essential for cell viability, *Mtbcpn60.1* is dispensable (Hu et al., 2008). The reason why *M. tuberculosis* has two *cpn60* genes is unclear. One view is that the MtbCpn60.2 protein functions as the housekeeping chaperonin and MtbCpn60.1 has evolved specialised functions, for example as a signalling protein. Another view is that both MtbCpn60.1 and MtbCpn60.2 function as molecular chaperones but have evolved to fold different clients and/or function under different cellular conditions (Fan et al., 2012; Kong et al., 1993; Lund, 2009; Mande & Kumar, 2017; Rao & Lund, 2010). Reports of their moonlighting properties as potent signalling molecules (Henderson et al., 2010) and evidence of their extracellular secretion (Cehovin et al., 2010; Hickey et al., 2009) complicate the understanding of their physiological functions. The fact that both proteins have potential roles in various aspects of Mycobacterial pathogenesis, such as granuloma formation (Hu et al., 2008), adhesion with macrophage cell surface receptors (Hickey et al., 2009, 2010) and blocking mitochondrial apoptosis pathways in infected macrophages (Joseph et al., 2017), is an important justification for an improved understanding of their cellular roles.

Structural and biophysical characterisation of MtbCpn60.1 and MtbCpn60.2 showed that their recombinant versions, expressed in and purified from *E. coli*, presented as lower order oligomers that failed to refold model proteins in standard in vitro chaperonin‐assisted protein refolding assays (Qamra & Mande, 2004; Qamra et al., 2004). On the contrary, MtbCpn60.1 has been shown to assemble as heptamers, tetradecamers and lower oligomers in *M. tuberculosis* (Kumar et al., 2009). Moreover, MtbCpn60.2 can complement for the loss of GroEL when expressed at sufficiently high levels and can also assemble into tetradecamers under some conditions (Fan et al., 2012; Hu et al., 2008). It is possible that the different oligomeric forms exist in a dynamic equilibrium, with the stress‐induced increase in chaperonin expression shifting the equilibrium towards higher‐order oligomers, including the canonical tetradecamers (Chilukoti et al., 2016; Fan et al., 2012). Post‐translational modifications (Canova et al., 2009; Kumar et al., 2009) and variations in cell physiology, such as altered local salt and nucleotide concentrations (Fan et al., 2012), might stabilise chaperonin tetradecamers and enable complete chaperone function. Therefore, we hypothesise that both MtbCpn60.1 and MtbCpn60.2 may potentially function as intracellular molecular chaperones.

The in vivo studies cited above assessed chaperone function of MtbCpn60.1 and MtbCpn60.2 by evaluating their ability to restore cell growth in *E. coli* depleted of GroEL. Because GroEL‐assisted protein folding of multiple chaperonin clients is essential for cell growth, this approach does not allow the detection of partial chaperone function (prevention of client aggregation without enhancing folding yield), or chaperone function that caters to distinct client subgroups. Therefore, we used additional assays to look for evidence of chaperone function of MtbCpn60.1 and MtbCpn60.2 in vivo, including their ability to assist the folding of GroEL clients, DapA, FtsE and MetK, under conditions of GroEL depletion. We demonstrate that MtbCpn60.2 is largely able to replace GroEL function in *E. coli* due to its ability to assist the folding of key GroEL clients. Moreover, we demonstrate that MtbCpn60.1, although unable to fully restore chaperonin activity in GroEL‐deficient *E. coli*, exhibits molecular chaperone function.

## 2 RESULTS

### 2.1 Expression of the *M. tuberculosis* chaperonin genes in GroEL‐depleted *E. coli*

Here, we probed the in vivo molecular chaperone functions of the *M. tuberculosis* Cpn60 proteins, MtbCpn60.1 and MtbCpn60.2, expressed in the *E. coli* MGM100 strain. MGM100 is a conditional *groEL* expression strain, wherein the chromosomal *groES‐groEL* operon is regulated by the arabinose‐inducible *P*_*BAD*_ promoter (McLennan & Masters, 1998). Therefore, MGM100 cells exhibit arabinose‐dependent GroEL production (Figure S1a) and fail to grow on glucose‐supplemented media unless a complementing chaperonin gene is expressed (McLennan & Masters, 1998). To assess the chaperone functions of MtbCpn60.1 and MtbCpn60.2, the chaperonin genes were cloned downstream of an IPTG‐inducible *P*_*trc*_ promoter and their cognate *Mtbcpn10* gene in pTrc99a (Amann et al., 1988; Table 1). We constructed chaperonin expression strains (Table 2) by transforming MGM100 cells with the chaperonin expression plasmids (Figure S1b). We then tested the ability of MtbCpn60.1 and MtbCpn60.2 to restore cellular chaperonin capacity in GroEL‐depleted *E. coli* MGM100 cells. For this, the strains were grown on glucose‐supplemented LB medium (LB‐glucose) for 2 h to deplete endogenous GroES and GroEL, and the plasmid‐borne chaperonin genes were induced with IPTG (Figure S1c). We confirmed overexpression of the chaperonin genes on SDS‐polyacrylamide gels and Western blots (Figure S2).

**TABLE 1:** Plasmids used in this study.

Plasmid	Description	Reference
pTrc99a	IPTG‐inducible expression vector, Amp^R^	Amann et al. (1988)
pEcoESL	pTrc99a‐based chaperonin expression plasmid carrying the *Escherichia coli groES‐groEL* operon [pTrc99a‐*Ecoli*‐*groES‐groEL*]	Hu et al. (2008)
pMtbCpn60.1	pTrc99a‐based chaperonin expression plasmid carrying the *M. tuberculosis cpn10‐cpn60.1* operon [pTrc99a‐*Mtb‐cpn10‐cpn60.1*]	Hu et al. (2008)
pMtbCpn60.2	pTrc99a‐based chaperonin expression plasmid carrying the *Mycobacterium tuberculosis cpn10* and *cpn60.2* genes in an operonic arrangement [pTrc99a‐*Mtb‐cpn10‐cpn60.2*]	Hu et al. (2008)

**TABLE 2:** Strains used in this study.

Strain	Description	Reference
*Escherchia coli* K‐12 substr. MG1655	K‐12 (λ‐) *rph‐1*	Guyer et al. (1981)
*Escherchia coli* K‐12 substr. MGM100	MG1655 *araBADp‐groE zje‐2335::kan*	McLennan and Masters (1998)
*Chaperonin expression strains* ^a^		
V	*Escherichia coli* MGM100 strain harbouring the control plasmid pTrc99a [MGM100(pTrc99a)]	This work
E	*Escherichia coli* MGM100 strain harbouring the *groES‐groEL* expression plasmid pEcoESL [MGM100(pEcoESL)]	This work
C1	*Escherichia coli* MGM100 strain harbouring the *Mtb‐cpn10‐cpn60.1* expression plasmid pMtbCpn60.1 [MGM100(pMtbCpn60.1)]	This work
C2	*Escherichia coli* MGM100 strain harbouring the *Mtb‐cpn10‐cpn60.2* expression plasmid pMtbCpn60.2 [MGM100(pMtbCpn60.2)]	This work

^a^ Strains constructed by transforming the *E. coli* MGM100 strain with the chaperonin expression plasmids (Table 1).

### 2.2 MtbCpn60.1 and MtbCpn60.2 suppress the cell filamentation phenotype caused by GroEL depletion

**FIGURE 1:**
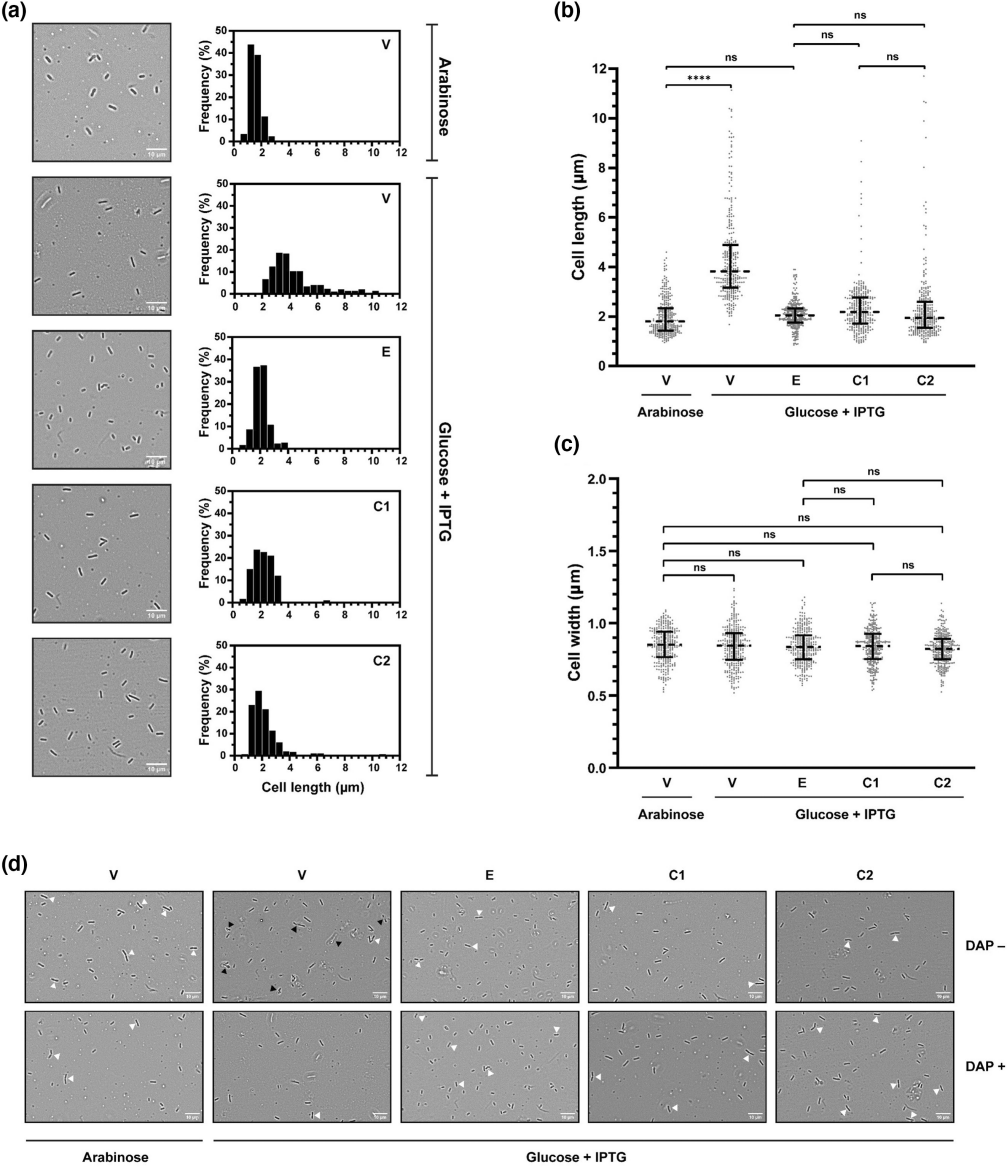
Effect of *Mycobacterium tuberculosis* chaperonins on cell size, division and lysis. Overnight cultures of chaperonin expression strains (V, E, C1, C2) were sub‐cultured in LB‐glucose and LB‐glucose‐DAP and grown alongside GroEL‐normal (V/Arabinose) cultures. At OD_600_ of 0.4–0.5 units, the LB‐glucose and LB‐glucose‐DAP cultures were induced with 100 μM IPTG and incubated at 37°C for 5 h. Culture aliquots were collected, diluted 100‐fold and examined by phase‐contrast microscopy for cell phenotypes. Cell lengths and widths were measured using the line measurement tool in Fiji image processing software (Schindelin et al., 2012) and subjected to statistical analyses using GraphPad Prism (www.graphpad.com). (a) Representative micrographs at 1000× following 5 h of growth in LB‐glucose‐DAP‐IPTG medium. Histograms show frequency distributions of cell lengths quantified from 300 cells of each strain. (b) Scatterplots showing the distribution of cell lengths. (c) Scatterplots showing the distribution of cell widths. (d) Micrographs representative of at least five fields of view, imaged at 1000× following 5 h of growth in LB‐glucose‐IPTG and LB‐glucose‐DAP‐IPTG media. Arrowheads indicate evidence of cell division (white) and lysis (black). Data: Scatterplots indicate median (‐‐‐‐) and interquartile range (error bars) of cell length and width. Statistical significance at *p* < 0.05 (Kruskal–Wallis test with Dunn's post hoc test). ****p < 0.0001; ns *p* > 0.05. Lanes: V—MGM100(pTrc99a), E—MGM100(pEcoESL), C1—MGM100(pMtbCpn60.1), C2—MGM100(pMtbCpn60.2).

GroEL deficiency causes filamentous growth in *E. coli* due to septation defects and impaired cell division arising from misfolding and loss of the obligate client FtsE, a cell‐division protein involved in septal‐ring assembly (Fujiwara & Taguchi, 2007; Fujiwara et al., 2010; Kerner et al., 2005). Consistent with this, we observed cell filamentation in GroEL‐depleted *E. coli* cells, with the cells appearing elongated. We confirmed this by imaging GroEL‐normal and GroEL‐depleted cells, and measuring cell lengths and widths. While their median cell widths were comparable, we determined GroEL‐depleted cells to have a median cell length of 4.2 μm, which approximates to a twofold increase from a median cell length of 1.8 μm estimated for GroEL‐normal cells (Figure S3). Overexpression of *groES‐groEL* from a complementing plasmid suppressed cell filamentation and reduced the median cell length to 2.0 μm, comparable to that observed for GroEL‐normal cells (Figure 1). We then imaged cells overexpressing *M. tuberculosis* chaperonins and measured their cell lengths and widths. We found that MtbCpn60.1 and MtbCpn60.2 reduced the median cell length to 2.2 and 1.9 μm respectively, which were comparable to GroEL‐normal and GroEL‐overexpressing cells (Figure 1b). We also observed increased frequency of cell division amongst cells overexpressing *groEL*, *Mtbcpn60.1* or *Mtbcpn60.2* (Figure 1d). Together, these results suggest that MtbCpn60.1 and MtbCpn60.2 sustain FtsE function by assisting its folding in GroEL‐depleted cells.

### 2.3 MtbCpn60.2, but not MtbCpn60.1, sustains proliferation of GroEL‐depleted *E. coli* cells

**FIGURE 2:**
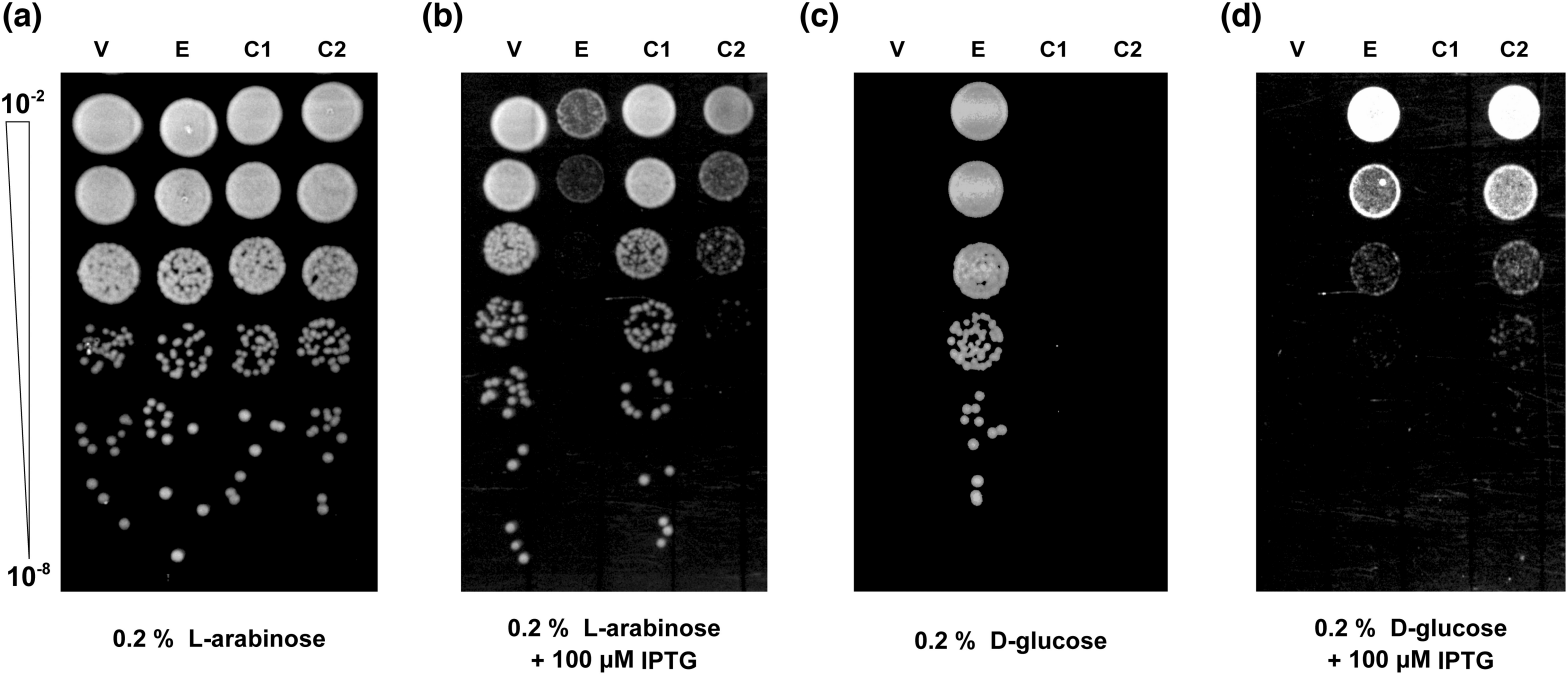
Role of *Mycobacterium tuberculosis* chaperonins in cell viability. Representative observations of the plate‐based cell growth assays at 30°C. The chaperonin expression strains were grown under GroEL‐depletion conditions for 2 h. The cultures were normalised for OD_600_, subjected to 10‐fold serial dilutions (from 10^−2^ to 10^−8^) and spotted onto LB‐agar plates supplemented with (a) 0.2% l‐arabinose, (b) 0.2% l‐arabinose + 100 μM IPTG, (c) 0.2% d‐glucose, and (d) 0.2% d‐glucose + 100 μM IPTG. Images representative of three independent experiments. Lanes: V—MGM100(pTrc99a), E—MGM100(pEcoESL), C1—MGM100(pMtbCpn60.1), C2—MGM100(pMtbCpn60.2).

The essential nature of multiple obligate GroEL clients explains why GroEL depletion in *E. coli* prevents growth on both solid and liquid media. We confirmed these phenotypes by assessing colony formation on agar plates and growth in LB medium (Figure S4), which were reversed upon overexpression of *groES* and *groEL* from a plasmid (Figure 2d). To evaluate the chaperone function of *M. tuberculosis* chaperonins, we examined their ability to support *E. coli* growth on plates and in liquid cultures under conditions of GroEL depletion.

We evaluated the growth phenotype on plates at 30, 37 and 42°C, with the results summarised in Table 3 (results at 30°C are shown in Figure 2 as a representative example). As expected, we observed normal cell growth and colony formation under GroEL‐normal conditions, due to expression of the endogenous *groES‐groEL* operon (Figure 2a). Upon suppression of the endogenous *groES‐groEL* operon, colony growth was observed for cells overexpressing *Mtbcpn10* and *Mtbcpn60.2* from the plasmid pMtbCpn60.2 (Figure 2d). However, MtbCpn60.2 complemented for the loss of GroEL at 30 and 37°C, but not at 42°C (Table 3). In contrast, no colony growth was observed for *Mtbcpn60.1* expressing cells under any conditions (Figure 2d and Table 3). These observations are in agreement with previous reports on MtbCpn60.1 and MtbCpn60.2 (Chilukoti et al., 2016; Fan et al., 2012; Hu et al., 2008), and are consistent with the hypothesis that MtbCpn60.2 is the canonical *M. tuberculosis* chaperonin. Interestingly, we observed colony growth for cells harbouring pEcoESL even on glucose‐supplemented plates that lack IPTG (Figure 2c). We found that the amount of GroEL produced due to the leakiness of the *P*_*trc*_ promoter (Figure S2d) was sufficient to support colony growth. Furthermore, we found high‐level overexpression of *groEL* (Figure 2b and Figure S5), and co‐overexpression of *groEL* and *Mtbcpn60.2* (Figure 2b) to be lethal, which is likely due to the fitness costs associated with chaperonin overproduction (Kumar et al., 2021; Sabater‐Muñoz et al., 2015).

**TABLE 3:** Summarised results of the colony formation assay probing *Escherichia coli* cell viability in the presence of *Mycobacterium tuberculosis* chaperonins.

Chaperonin	Co‐chaperonin	Incubation temperature		
		30°C	37°C	42°C
−	−	−	−	−
GroEL^a^	GroES^a^	+++	+++	+++
GroEL^b^	GroES^b^	+	+	+
MtbCpn60.1	MtbCpn10	−	−	−
MtbCpn60.2	MtbCpn10	+++	++	−
GroEL^a^ + MtbCpn60.1	GroES^a^ + MtbCpn10	+++	+++	−
GroEL^a^ + MtbCpn60.2	GroES^a^ + MtbCpn10	+	+	−

*Note*: The table summarises the observations from the plate‐based complementation assay on LB‐agar plates supplemented with either 0.2% l‐arabinose or 0.2% d‐glucose, in the presence or absence of 100 μM IPTG (added to induce the plasmid‐borne chaperonin genes). A representative image is shown in Figure 2. The plates were analysed for colony growth, which was then correlated with chaperonin expression. +++ represents colony growth similar to the positive control, ++ represents fewer colony numbers, + represents fewer colony numbers with small colonies, − represents no growth.

^a^ GroES‐GroEL expression from plasmid only.

^b^ GroES‐GroEL expression from chromosome and plasmid.

**FIGURE 3:**
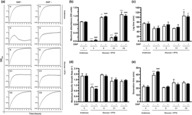
Role of *Mycobacterium tuberculosis* chaperonins in cell growth and proliferation. Overnight cultures of the chaperonin expression strains (V, E, C1, C2) were sub‐cultured in LB‐glucose and LB‐glucose‐DAP media and grown in parallel with the GroEL‐normal (V/Arabinose DAP− and V/Arabinose/DAP+) cultures of the *Escherichia coli* MGM100(pTrc99a) strain. Sub‐cultures with absorbance (OD_600_) of 0.4–0.5 were diluted 100‐fold into LB‐glucose‐IPTG and LB‐glucose‐DAP‐IPTG media and incubated at 37°C, with absorbance measured at 10 min intervals to monitor culture growth. (a) Semi‐log plots illustrating growth curves of chaperonin expression strains. Data points represent mean absorbance of three biological replicates plotted on a logarithmic *y*‐axis, and curves represent best fit of the Gompertz growth model. (b–e) Representative bar graphs illustrating growth parameters estimated for each of the cultures. Data presented as mean ± SEM of three biological replicates. (b) Maximum absorbance depicts maximum OD_600_ for each of the cultures. (c) Lag time depicts the duration of lag phase before the cultures progressed to exponential growth. (d) Maximum specific growth rate depicts the highest specific growth rate achieved during exponential growth. (e) Generation time depicts the average doubling time observed during exponential growth. Data: Statistical significance at *p* < 0.05 (one‐way ANOVA with Bonferroni post hoc test). ****p < 0.0001, **0.001 < *p* < 0.01 compared to V/arabinose/DAP−; ^####^ *p* < 0.0001, #0.01 < *p* < 0.05 compared to V/arabinose/DAP+. Lanes: V—MGM100(pTrc99a), E—MGM100(pEcoESL), C1—MGM100(pMtbCpn60.1), C2—MGM100(pMtbCpn60.2).

To study the growth phenotype in liquid medium, we compared the growth kinetics of the chaperonin expression strains at 37°C under conditions of GroEL depletion (Figure 3). Growth parameters were estimated by fitting the growth curves using the Gompertz model of logistic growth (Gompertz, 1825; Zwietering et al., 1990). Growth of *Mtbcpn60.2* expressing cells was broadly similar to that of *groEL* expressing cells, although with longer lag times (Figure 3). This suggested that MtbCpn60.2 functions in a manner similar to GroEL, when it is expressed in *E. coli*. Besides, overexpression of either *groEL* or *Mtbcpn60.2* enhanced *E. coli* culture yield as indicated by approximately 1.5‐fold higher absorbance of stationary phase cultures relative to GroEL‐normal cells (Figure 3b). In contrast, cultures of *Mtbcpn60.1* expressing cells grew only for a brief period of about 2 h before proceeding into an early and prolonged stationary phase (Figure 3a) with culture yield reduced to approximately 0.2‐fold relative to GroEL‐normal cells (Figure 3b).

### 2.4 MtbCpn60.1 and MtbCpn60.2 prevent lysis of GroEL‐depleted *E. coli* cells

GroEL‐depleted *E. coli* cells undergo lysis, which results in a decline in the absorbance of liquid cultures after a brief period of growth (McLennan & Masters, 1998). We observed a similar decline in the absorbance of cultures of GroEL‐depleted *E. coli* cells in our growth kinetics experiments (Figure 3a), which we confirmed to be an outcome of cell lysis by observing cells under a phase contrast microscope (Figure 1d). The cell lysis phenotype is a known consequence of low cellular levels of meso‐2,6‐diaminopimelic acid (DAP) (McLennan & Masters, 1998), which is an essential component of cell wall peptidoglycans and is synthesised by the DAP sub‐pathway enzymes (DapA‐DapE) in the l‐lysine biosynthetic pathway (Scapin & Blanchard, 1998). DapA, the first enzyme of the DAP sub‐pathway, is an obligate GroEL client (Kerner et al., 2005). Therefore, GroEL depletion results in misfolding and depletion of DapA, leading to low DAP levels, failure of cell wall synthesis and eventually cell lysis (McLennan & Masters, 1998). We confirmed that DAP supplementation prevented cell lysis (Figure 1d) and restored growth (Figure 3a) in GroEL‐depleted cells. Overexpression of plasmid‐borne chaperonins, *groES‐groEL*, *Mtb‐cpn10‐cpn60.1* or *Mtb‐cpn10‐cpn60.2*, substantially reduced the frequency of cell lysis (Figure 1d) and restored culture growth (Figure 3a) in the absence of DAP. Further, the addition of DAP to cultures of the chaperonin expression strains did not improve their growth (Figure 3), suggesting that chaperonin overexpression restored cellular growth by maintaining DAP at levels sufficient to prevent cell lysis. Together, these results suggest that GroEL, MtbCpn60.1 and MtbCpn60.2 have similar effects on the intracellular levels of DAP, which they sustain by stabilising DapA and assisting its folding and hence supporting its function.

### 2.5 MtbCpn60.1 prevents MetK aggregation, while MtbCpn60.2 improves the yield of functional MetK

We further investigated the intracellular activities of MtbCpn60.1 and MtbCpn60.2 using another known chaperonin client. A suitable candidate was S‐adenosylmethionine synthase (MetK), an enzyme encoded by the *metK* gene of the *met* regulon, which catalyses the formation of S‐adenosylmethionine (SAM) from methionine and ATP (Markham et al., 1980). Moreover, MetK is an obligate GroEL client (Kerner et al., 2005), and hence, GroEL depletion results in its misfolding, aggregation and consequential depletion (Chapman et al., 2006; Fujiwara & Taguchi, 2012; Kerner et al., 2005). Low levels of functional MetK result in reduced cellular levels of SAM and de‐repression of the *met* regulon, including *metE*, which encodes an 85 kDa B_12_‐independent methionine synthase (Fujiwara & Taguchi, 2012). Thus, overproduction of MetE indicates loss of MetK function due to GroEL deficiency (Chapman et al., 2006; Fujiwara & Taguchi, 2012; Horwich et al., 1993).

**FIGURE 4:**
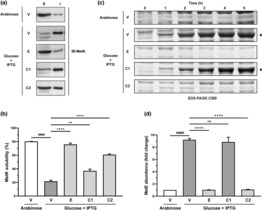
Effect of *Mycobacterium tuberculosis* chaperonins on MetK folding and function. Cultures of chaperonin expression strains (V, E, C1, C2) were grown in LB‐arabinose or LB‐glucose‐DAP medium. At OD_600_ of 0.4–0.5 units, glucose cultures were induced with 100 μM IPTG for 5 h to express plasmid‐borne chaperonins. (a) Prevention of MetK aggregation. Cells were harvested after 5 h of IPTG induction, followed by extraction of total protein, fractionation into soluble (S) and insoluble (I) fractions, resolution of the fractions on SDS‐polyacrylamide gels and immunoblotting (IB) with anti‐MetK antibody. (b) Relative MetK solubility. The immunoblots were subjected to relative densitometric analysis to compute the percentage of MetK obtained in the soluble fraction. The graph shows mean relative MetK solubility observed in the presence of the indicated chaperonin homologues. (c) Enhancing of MetK folding and function. Culture aliquots were collected at 1 h intervals after IPTG induction, followed by extraction of total protein, resolution of the extracts on SDS‐polyacrylamide gels and Coomassie Brilliant Blue (CBB) staining of the gels to observe the levels of MetE. The solid arrows indicate MetE protein bands. (d) Relative MetE abundance as a measure of MetK functional state. Culture samples were collected at 0 and 5 h after IPTG induction and cells harvested, followed by extraction of total protein, resolution of the extracts on SDS‐polyacrylamide gels, and CBB staining of the gels to observe MetE overexpression. The gels were subjected to relative densitometric analysis to estimate the change in cellular levels of MetE upon chaperonin expression. Fold change in MetE abundance was computed relative to the GroEL‐normal MGM100(pTrc99a) strain (V/arabinose). The graph shows normalised MetE abundance observed in the presence of the chaperonin homologues as indicated. Data: All images and data representative of three independent experiments. Bar graphs present data as Mean ± SEM. Statistical significance determined at *p* < 0.05 (one‐way ANOVA followed by the Bonferroni post hoc test for multiple comparisons). ****p < 0.0001, **0.001 < *p* < 0.01, ns *p* > 0.05 compared to V/glucose; ^####^ *p* < 0.0001 compared to V/arabinose. Lanes: V—MGM100(pTrc99a), E—MGM100(pEcoESL), C1—MGM100(pMtbCpn60.1), C2—MGM100(pMtbCpn60.2).

We first confirmed the correlation between GroEL depletion, MetK aggregation and MetE overproduction (Figure S6). Approximately 80% of MetK was found in the soluble fraction of GroEL‐normal cell lysates. GroEL depletion reduced the levels of soluble MetK by fourfold to approximately 20% (Figure S6b) and increased the cellular levels of MetE by approximately ninefold (Figure S6c). Overexpression of *groES‐groEL* from a complementing plasmid restored MetK folding and function in GroEL‐depleted cells, with MetK solubility increased to approximately 80% (Figure 4a,b) and MetE levels equivalent to those observed in GroEL‐normal cells (Figure 4c,d).

We then investigated the chaperone function of *M. tuberculosis* chaperonins by testing their ability to suppress MetK aggregation and enhance the yield of functional MetK. Cells overexpressing *Mtbcpn60.1* showed an increase in soluble MetK to approximately 40% (Figure 4a,b), while the cellular levels of MetE were comparable to GroEL‐depleted cells (Figure 4c,d). The observation of ninefold higher‐than‐normal MetE levels indicates that, despite a twofold increase in MetK solubility, cells overproducing MtbCpn60.1 could not sustain normal SAM levels due to the lack of functional MetK. On the contrary, overexpression of *Mtbcpn60.2* increased MetK solubility to approximately 60% (Figure 4a,b) and reduced MetE levels to that observed in GroEL‐normal cells (Figure 4c,d). Therefore, MtbCpn60.2 not only improved MetK solubility by approximately threefolds but also restored normal cellular levels of SAM and MetE by enhancing the yield of correctly folded and functional MetK. These results suggest that, of the two *M. tuberculosis* chaperonins, MtbCpn60.1 is only capable of preventing MetK aggregation, while MtbCpn60.2 assists the folding of MetK into its functional state.

## 3 DISCUSSION

In this study, we examined the intracellular function of the *M. tuberculosis* chaperonin paralogs, *Mtbcpn60.1* and *Mtbcpn60.2*, using *E. coli* cell‐based assays that enable tracking of the folding states of the endogenous obligate chaperonin clients, FtsE, DapA and MetK. We demonstrated the molecular chaperone function of the enigmatic *M. tuberculosis* chaperonin protein, MtbCpn60.1, which had eluded chaperone biologists thus far. In addition, we demonstrated the protein folding activity of the essential paralog, MtbCpn60.2, and extended the existing knowledge about its molecular chaperone function. Our results show that despite exhibiting potential functional divergence (Goyal et al., 2006; Mande et al., 2013), both MtbCpn60.1 and MtbCpn60.2 exhibit intracellular molecular chaperone function.

MtbCpn60.1, MtbCpn60.2 and GroEL had similar effects on cell growth with regard to the similarities in culture growth rates and generation times irrespective of which chaperonin was expressed (Figure 3). Complementation with MtbCpn60.1 or MtbCpn60.2 effectively (a) reversed the septation defects and cell filamentation phenotype caused by GroEL depletion and restored normal cell size and (b) enabled normal cell division and morphology (Figure 1), suggesting that MtbCpn60.1 and MtbCpn60.2 supported FtsE folding and normal septal ring formation during cell division. MtbCpn60.1 and MtbCpn60.2 had comparable effects in preventing the lysis of GroEL‐depleted *E. coli* cells in the absence of DAP (Figures 1d and 3a). This suggested that both MtbCpn60.1 and MtbCpn60.2, like GroEL, maintain intracellular DAP levels by assisting DapA folding.

Despite these similarities, we observed specific functional differences between GroEL, MtbCpn60.1 and MtbCpn60.2. Cells overexpressing *Mtbcpn60.1* stopped dividing and entered the stationary phase prematurely, showing that MtbCpn60.1 could not support exponential culture growth beyond 2 h, unlike GroEL and MtbCpn60.2 (Figure 3). Moreover, while MtbCpn60.2 improved the yield of soluble MetK (Figure 4a,b) and effectively restored MetK function (Figure 4c,d), MtbCpn60.1 had limited ability to suppress MetK aggregation (Figure 4a,b) or support MetK folding and function (Figure 4c,d). This suggested that MtbCpn60.2 assists the folding of nascent MetK polypeptides into their functional states. We speculate that, in the presence of MtbCpn60.1, nascent MetK polypeptides remain soluble but misfolded and hence are non‐functional. The turnover of soluble MetK was significantly lower with MtbCpn60.1 than MtbCpn60.2 or GroEL, possibly due to relatively lower expression levels of the former (Figure S2). In *E. coli*, at least 13 essential proteins, including DapA and MetK, depend on GroEL for their folding and conformational maintenance (Kerner et al., 2005). Our results suggest that MtbCpn60.1 does not effectively assist the folding of all the obligate chaperonin clients that are essential for key cellular processes like growth, metabolism and proliferation. This would explain the low fitness, the premature growth arrest in liquid media and the lack of growth on solid media of the cells overexpressing *Mtbcpn60.1*. A key difference between MtbCpn60.2 and GroEL was the failure of the former to sustain growth at higher temperatures (Table 3). This has been observed before with heterologous chaperonins in *E. coli* (Fan et al., 2012; Ivic et al., 1997) and may be related to the increased demand for chaperone function at higher temperatures caused by the need to stabilise more proteins under temperature stress (Balchin et al., 2016). The lower expression (Figure S2) and thermostability (Qamra et al., 2004) of MtbCpn60.2 compared to GroEL likely result in reduced cellular fitness at higher temperatures.

Our findings are consistent with the hypothesis that the *M. tuberculosis* chaperonin proteins, MtbCpn60.1 and MtbCpn60.2, are intracellular molecular chaperones. While MtbCpn60.2 exhibits GroEL‐like protein folding activity, MtbCpn60.1 also chaperones client proteins by increasing their conformational stability and preventing their aggregation. The functional differences between GroEL, MtbCpn60.1 and MtbCpn60.2 may be a consequence of sequence differences. MtbCpn60 proteins have greater than 50% identity and greater than 65% similarity with GroEL (Figure S7). MtbCpn60.2 exhibits greater similarity to GroEL (73.2%) than MtbCpn60.1 (67.5%), which may explain the greater functional divergence of the latter. Sequence alignments show differences in the highly conserved equatorial domain residues, particularly at the N‐ and C‐termini (Figure S8). The inter‐subunit interactions that stabilise the GroEL tetradecamer occur mostly between equatorial domain residues, including key residues at the N‐terminus (Braig et al., 1994; Xu et al., 1997). Mutating the GroEL A2 (Horovitz et al., 1993) and E76 (Qamra et al., 2004) residues to serines, the corresponding residues in MtbCpn60.1, destabilised the GroEL tetradecamer. Similarly, swapping the 22 N‐terminal residues of MtbCpn60.2 with the equivalent sequence from GroEL enhanced the stability of the chimeric chaperonin tetradecamers (Fan et al., 2012). These observations are consistent with the lower thermodynamic stability of the recombinant MtbCpn60 proteins (Qamra et al., 2004), which may contribute to the inability of MtbCpn60.2 to function in *E. coli* at high temperatures. Likewise, the GroEL C‐terminus participates in client interaction and folding (Weaver & Rye, 2014). While MtbCpn60.2 has a GroEL‐like, glycine‐methionine‐rich hydrophobic C‐terminus, MtbCpn60.1 has a Histidine‐rich charged C‐terminus (Lund, 2009). Therefore, we speculate that the client pools of GroEL and MtbCpn60.2 might overlap to a large extent, while MtbCpn60.1 might interact with a specific (sub)set of clients, which may contribute to its proposed functional specialisation and reduced ability to function in *E. coli*.

Interestingly, we noted some evidence of cytotoxicity upon co‐expression of chromosomal *groEL* and plasmid‐borne *Mtbcpn60.2* (Figure 2b). The reduced growth of the cells expressing both chromosomal and plasmid‐borne copies *of groEL* (Figure 2b and Figure S5) suggests a fitness trade‐off associated with chaperonin overproduction. For optimal fitness, cells regulate chaperonin levels to maintain an economical balance in the rates of protein synthesis and folding (Santra et al., 2017). Excess chaperonins are likely to reduce cellular fitness by holding essential proteins through prolonged, non‐specific and often non‐productive binding (Badcoe et al., 1991; Kumar et al., 2021; Kumar & Mande, 2011), thereby reducing their availability for cellular housekeeping functions. Furthermore, chaperonin overproduction was previously shown to have high energetic (Sabater‐Muñoz et al., 2015) and metabolic (Kumar et al., 2021) costs and may have additional fitness costs associated with translational overload and reduced overall cellular protein synthesis capacity (Dong et al., 1995).

The interpretation of assay results can be complicated by the differences in expression levels of the chaperonin homologues (Chilukoti et al., 2016; Fan et al., 2012; Kumar et al., 2009). The cellular levels of the two Mycobacterial chaperonin proteins were significantly different, with MtbCpn60.2 being clearly visible on CBB‐stained SDS‐PAGE gels, whereas MtbCpn60.1 could only be detected by Western blotting (Figure S2). We have generally observed that Mycobacterial *cpn60.1* genes are poorly expressed in *E. coli* (Fan et al., 2012; Rao & Lund, 2010), and multiple approaches to improve their expression levels have not met with significant success. Interestingly, the difference in expression levels of the two chaperonins has also been observed in *M. tuberculosis* in both RNA‐seq and ribosome profiling analysis (Shell et al., 2015). Since we observed lower levels of MtbCpn60.1 compared to GroEL and MtbCpn60.2, it is possible that MtbCpn60.1 would support cell proliferation if expressed at sufficiently higher levels. However, the fact that cells expressing MtbCpn60.1 neither lyse nor exhibit septation defects shows that its levels under the tested conditions are sufficient to sustain DapA and FtsE in their correctly folded and functional states.

While we have previously reported the ability of MtbCpn60.2 to complement for the loss of GroEL in *E. coli* (Chilukoti et al., 2016; Fan et al., 2012; Hu et al., 2008), its protein folding activity could not be demonstrated with certainty. Herein, we provide evidence to establish the protein folding activity of MtbCpn60.2. Moreover, this is the first report showing the molecular chaperone function of Mycobacterial Cpn60.1 proteins. Whether the level of function of the *M. tuberculosis* chaperonins would improve with higher expression levels remains to be determined. Future studies focussed on mapping the client repertoires of the two MtbCpn60 proteins are necessary to impart clarity on whether these functional differences are due to their interaction with distinct client (sub)groups. Forthcoming studies focussed on the biochemical properties of MtbCpn60.1 and MtbCpn60.2 are underway, with the aim to shed light on the specifics of their functional mechanisms and modes of action. Finally, it remains to be seen if their roles as intracellular molecular chaperones are linked with their roles in Mycobacterial pathogenesis.

## 4 CONCLUSIONS

Our interest in *M. tuberculosis* chaperonins stems from their inability to assemble into higher‐order oligomers, under conditions where GroEL exists as a stable tetradecamer, and the evidence that they have key roles in the pathogenesis and progression of tuberculosis. Although MtbCpn60.2 has been demonstrated to functionally replace GroEL, MtbCpn60.1 function has been enigmatic. Our findings suggest that both MtbCpn60.1 and MtbCpn60.2 can act as molecular chaperones possibly with distinct clientomes or modes of action. The difference in activities may be due to intrinsic properties or may relate to the different levels of production of these two Cpn60 proteins. Future studies will focus on understanding their biochemical properties and mapping their client repertoires in their native host.

## 5 MATERIALS AND METHODS

### 5.1 Bacterial strains and plasmids

The *E. coli* MGM100 strain (McLennan & Masters, 1998) was a generous gift from Prof. Millicent Masters. pTrc99a‐based expression plasmids containing *E. coli* and *M. tuberculosis* chaperonin genes (Table 1) were introduced into *E. coli* MGM100 to construct chaperonin expression strains (Table 2).

### 5.2 Chemicals and reagents

All culture media, supplements, antibiotics and bovine serum albumin (BSA; #MB083) were purchased from HiMedia Laboratories. Buffers, salts, Protease Inhibitor Cocktail set II (#539132) and Meso‐2,6‐Diaminopimelic acid (DAP; #07036) were procured from Merck. The anti‐GroEL (#ADI‐SPS‐875) and anti‐MetK (#A1900) antibodies were purchased from Enzo Life Sciences and BioVision Inc., respectively. The anti‐MtbCpn60.1 antibody, used in Western blotting experiments to detect MtbCpn60.1, was raised against the unique Histidine‐rich carboxy terminus of MtbCpn60.1 (H_3_N^+^‐DKPAKAEDHDHHHGHAH‐COO^−^). This custom antibody (mAbCMS2) was synthesised at Pepceuticals Ltd. The HRP‐conjugated IgG secondary antibody (#7074) was purchased from Cell Signaling Technology. Pierce ECL Plus substrate for western blotting (#32134) was purchased from Thermo Fisher Scientific.

### 5.3 Bacterial culture conditions

*Escherichia coli* suspension cultures were grown in lysogeny broth (LB), and colony formation was examined on LB‐agar plates. Culture media were supplemented with 50 μg/mL kanamycin to select for MGM100 and 100 μg/mL ampicillin to select for the chaperonin expression plasmids (Table 1). Culture media were supplemented with l‐arabinose or d‐glucose at 0.2% (w/v), 100 μM IPTG and 100 μg/mL DAP as appropriate. Overnight cultures were grown from single colonies inoculated into LB‐arabinose medium. GroEL‐normal cultures were grown in LB‐arabinose medium from overnight cultures diluted to an initial absorbance of 0.05 OD_600_ units. To grow GroEL‐depleted cultures, GroEL‐normal cultures were pelleted by centrifugation, washed with fresh LB medium, normalised for OD_600_, used to inoculate LB‐glucose‐DAP medium to an initial absorbance of 0.05 OD_600_ units and incubated for 2 h. Culture media were supplemented with 100 μg/mL DAP to prevent cell lysis caused by GroEL depletion. All cultures were incubated at 37°C unless specified otherwise.

### 5.4 Microscopy

Overnight cultures of the chaperonin‐expression strains (Table 2) were sub‐cultured (0.05 OD_600_ units) into LB‐glucose and LB‐glucose‐DAP media. At OD_600_ of 0.4–0.5 units, the sub‐cultures were induced with 100 μM IPTG and incubated at 37°C for 5 h. Culture aliquots were collected, diluted 100‐fold and examined for the phenotypic features of cell lysis, division, morphology and size, at 1000× using a Nikon H600L microscope (Nikon Corporation) with a 100× oil‐immersion phase contrast objective. Cell lengths and widths were measured from the micrographs using the line measurement tool in Fiji image processing software (Schindelin et al., 2012). Strain‐specific differences in cell lengths and widths were assessed for statistical significance through the non‐parametric Kruskal–Wallis test, followed by Dunn's post hoc test for multiple comparisons.

### 5.5 Plate‐based complementation assay

Overnight cultures of the chaperonin expression strains (Table 2) were sub‐cultured (0.05 OD_600_ units) in LB‐glucose‐DAP medium. Cells were harvested in the mid‐log phase by centrifugation at 8000 *g*, resuspended in fresh LB medium to OD_600_ of 1.0 and serially diluted in 10‐fold steps. The dilutions (10^−1^ to 10^−8^) were spotted onto three sets of LB‐agar plates supplemented with either 0.2% (w/v) l‐arabinose or 0.2% (w/v) d‐glucose in the presence and absence of 100 μM IPTG. Each set was subsequently incubated at 30, 37 and 42°C and analysed for colony formation and morphology.

### 5.6 Growth kinetics assay

Overnight cultures of chaperonin expression strains (Table 2) were sub‐cultured into LB‐glucose and LB‐glucose‐DAP media at 1% (v/v) inoculum and grown to an OD_600_ of 0.4–0.5 units. Sub‐cultures were normalised for OD_600_, diluted (1:100) into fresh LB‐glucose‐IPTG and LB‐glucose‐DAP‐IPTG media and dispensed into the wells of a 96‐well plate. The plate was sealed with a sterile, gas‐permeable membrane and incubated at 37°C with continuous shaking at 350 cpm, in a BioTek Citation 5 Multi‐mode plate reader, and OD_600_ was measured at 10 min intervals. The OD_600_ values were plotted against time and fitted into the Gompertz model of logistic growth (Gompertz, 1825; Zwietering et al., 1990) using non‐linear regression analysis workflows in GraphPad Prism version 8.0.2 for Windows (GraphPad Software, www.graphpad.com). Logarithmic growth curves plotted from the exponential phase data were used to compute specific growth rates and generation times. The growth parameters (Table S1) of strains expressing *M. tuberculosis* chaperonins were compared with those of the strains expressing *groES‐groEL*, and differences were tested for statistical significance using one‐way ANOVA followed by the Bonferroni post hoc test for multiple comparisons.

### 5.7 Preparation and fractionation of total protein extracts

Cells were resuspended in ice‐cold lysis buffer (50 mM Tris–HCl, pH 8.0; 100 mM NaCl; 1× protease inhibitor cocktail) and lysed by ultrasonication using a Branson Sonifier S450A (Emerson Electric Co.) at 30% amplitude with alternating on and off pulses of 10 and 50 s, respectively. Cell lysates (total protein extracts) were separated into soluble and insoluble fractions by centrifugation at 15,000 *g* for 30 min. Total, soluble and insoluble protein extracts were subsequently analysed by SDS–polyacrylamide gel electrophoresis as described in Sambrook & Russel, 2001 and immunoblotting as described below.

### 5.8 Western blotting

Samples of total, soluble and insoluble protein fractions were resolved on 10% SDS–polyacrylamide gels and transferred to PVDF membranes by electroblotting. Membranes were blocked with 5% BSA and incubated with the appropriate primary antibodies (anti‐GroEL, anti‐MetK, or anti‐MtbCpn60.1) for 12–14 h, followed by 2 h incubation with HRP‐conjugated secondary antibody. Blots were developed with Pierce ECL Plus western blotting substrate and subsequently visualised on Typhoon FLA 9500 (GE Healthcare Life Sciences).

### 5.9 MetK solubility and function assay

Overnight cultures of the chaperonin expression strains (Table 2) were sub‐cultured (0.05 OD_600_ units) in LB‐glucose‐DAP medium. The cultures were grown until the OD_600_ reached 0.4–0.5 units and induced with 100 μM IPTG for 5 h. Subsequently, cells were harvested and lysed to extract total cellular protein. The total protein extracts were fractionated into soluble and insoluble fractions. To assess MetK solubility, equal volumes of the normalised soluble and insoluble protein fractions were resolved on an SDS‐polyacrylamide gel followed by immunoblotting with anti‐MetK antibody. MetK protein levels in the soluble and insoluble fractions were quantified by densitometric analysis of the immunoblots using Quantity One 1‐D Analysis Software (Bio‐Rad Laboratories Inc., USA). Solubility of fractionated MetK was computed as the percentage of protein that separated into the soluble fraction. To assess MetK function, equivalent amounts of total protein extracts were resolved on an SDS‐polyacrylamide gel followed by staining with Coomassie Brilliant Blue R‐250. Relative levels of MetE protein were quantified by densitometry as above. Differences in MetK solubility and MetE abundance were assessed for statistical significance using Student's unpaired *t* test with Welch's correction for pairwise comparisons, and one‐way ANOVA followed by the Bonferroni post hoc test for multiple comparisons.

### 5.10 Data collection and statistical analyses

All observations illustrate representative data collected through a minimum of three independent experiments. Quantitative data represent Mean ± SE of three or more biological replicates and were tested for statistical significance with *p* < 0.05 deemed significant. All statistical analyses were performed using GraphPad Prism version 8.0.2 for Windows (GraphPad Software, www.graphpad.com).

## AUTHOR CONTRIBUTIONS

Bakul Piplani: Conceptualization, Methodology, Investigation, Formal analysis, Validation, Visualisation, Writing—Original draft preparation, Writing—Reviewing and Editing, Funding acquisition. C. M. Santosh Kumar: Conceptualization, Methodology, Visualisation, Writing—Reviewing and Editing, Funding acquisition. Peter A. Lund: Conceptualization, Resources, Methodology, Visualisation, Writing—Reviewing and Editing, Supervision, Funding acquisition. Tapan K. Chaudhuri: Conceptualization, Resources, Writing—Reviewing and Editing, Supervision, Project administration, Funding acquisition.

## CONFLICT OF INTEREST STATEMENT

The authors declare that they have no conflicts of interests.

## ETHICS STATEMENT

This article does not contain any studies with human participants or animals.

## Supporting information


Figure S1.

## Data Availability

The data that support the findings of this study are available from the corresponding author upon reasonable request.
