# Supplementary material for: Mycobacterial chaperonins in cellular proteostasis: Evidence for chaperone function of Cpn60.1 and Cpn60.2‐mediated protein folding
Source: Mol Microbiol. 2023 Jun 23;120(2):210–23. doi: 10.1111/mmi.15109 (PMC10952152; doi:10.1111/mmi.15109)
Supplement: Supplementary file 1 — Figure S1. [file MMI-120-210-s001.pdf]

Supplementary Information

Title:

Mycobacterial chaperonins in cellular proteostasis: Evidence for chaperone function of Cpn60.1 and Cpn60.2-mediated protein folding

Short/Running Title:

Chaperone functions of *M. tuberculosis* chaperonins

Authors:

Bakul Piplani[1], C. M. Santosh Kumar[2,3], Peter A. Lund[2,3], Tapan K. Chaudhuri[1*].

Affiliations:

[1] Kusuma School of Biological Sciences, Indian Institute of Technology Delhi, Hauz Khas, New Delhi, India

[2] School of Biosciences, University of Birmingham, Edgbaston, Birmingham, UK

[3] Institute of Microbiology and Infection, University of Birmingham, Edgbaston, Birmingham, UK

*Correspondence: Dr. Tapan K. Chaudhuri, Kusuma School of Biological Sciences, Indian Institute of Technology Delhi, New Delhi 110016, India; email: tkchaudhuri@bioschool.iitd.ac.in

Supplementary Figures

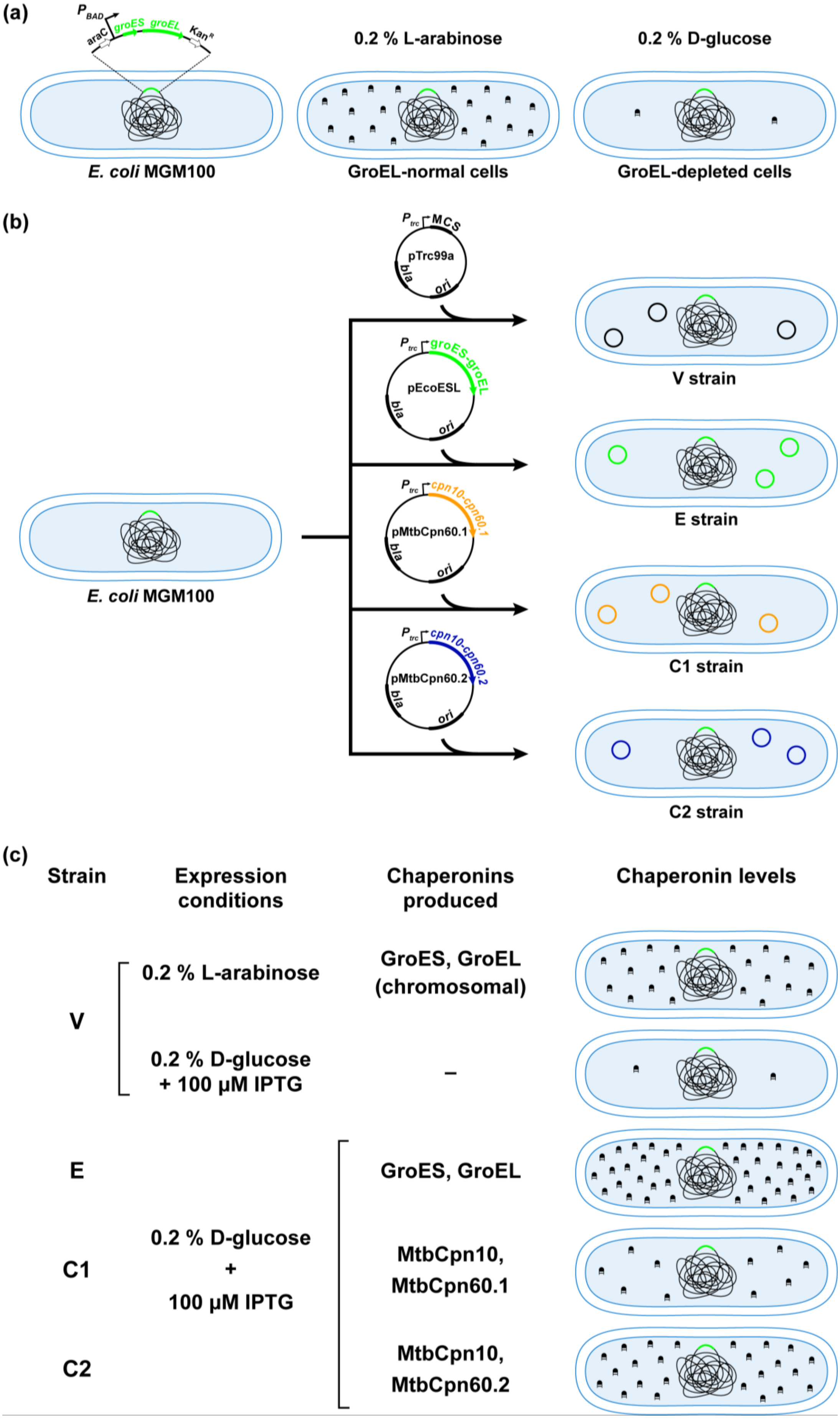

Figure S1: Schematic illustration of the chaperonin expression strains, expression conditions and cellular chaperonin levels (adapted from Kumar *et al.*, 2021).

(a) Schematic illustrating GroEL-normal and GroEL-depleted cells of the *E. coli* MGM100 strain. In MGM100, the chromosomal *groES-groEL* operon is regulated by the arabinose-inducible P_{BAD} promoter (McLennan and Masters, 1998). GroEL production in MGM100 cells requires growth in arabinose-supplemented medium, and growth in glucose-supplemented medium is accompanied by an approximately 90 % reduction in cellular GroEL levels (McLennan and Masters, 1998). GroES-GroEL complexes (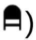) are shown to indicate cellular levels of GroES and GroEL.

(b) Schematic illustrating construction of the chaperonin expression strains. Strains V, E, C1 and C2 were constructed by transforming the *E. coli* MGM100 strain with chaperonin expression plasmids, pTrc99a (black), pEcoESL (green), pMtbCpn60.1 (orange) and pMtbCpn60.2 (blue), respectively.

(c) Schematic illustrating the growth conditions for chaperonin production and the corresponding levels of chaperonins produced in each strain. The strains were grown in glucose-supplemented growth medium to repress the chromosomal *groES-groEL* operon and the plasmid-borne chaperonin genes were induced with 100 μM IPTG. Cellular chaperonin levels were highest in the GroES-GroEL producing E strain, followed by the MtbCpn10-MtbCpn60.2 producing C2 strain, and the lowest in the MtbCpn10-Cpn60.1 producing C1 strain. Chaperonin complexes (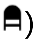) are shown in each strain to indicate cellular levels of the chaperonin proteins (GroES/MtbCpn10 and GroEL/MtbCpn60.1/MtbCpn60.2).

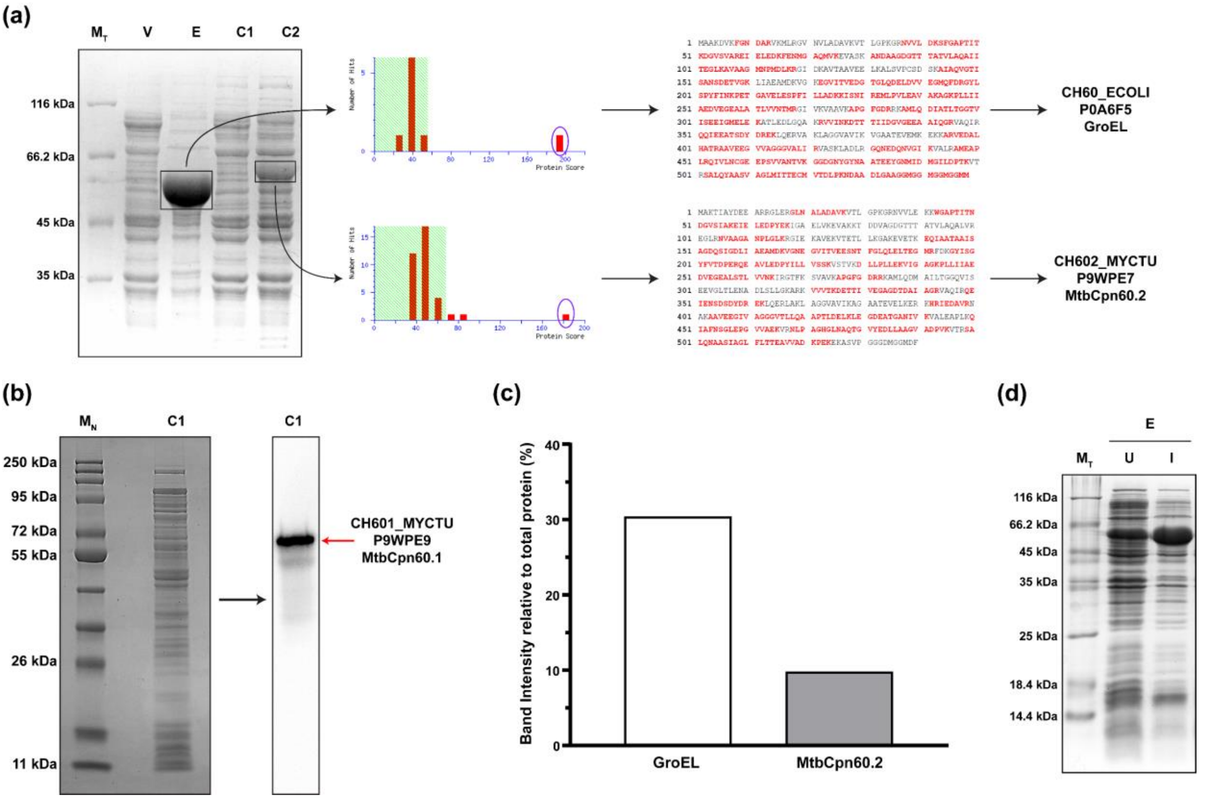

Figure S2: Expression of plasmid-borne chaperonin genes in *E. coli* MGM100.

Overnight cultures of the chaperonin expression strains (V, E, C1 and C2) were sub-cultured in LB-glucose-DAP medium, grown to 0.4 – 0.5 OD_{600} units and induced with 100 μM IPTG for 5 h. Cells were harvested by centrifugation, and total protein extracts were prepared and resolved on 10% SDS-polyacrylamide gels. The gels were stained with Coomassie Brilliant Blue R-250 (CBB) and analysed for chaperonin production.

(a) MS analysis of chaperonin proteins. Overexpressed protein bands corresponding to ~ 60 kDa were excised, digested with Trypsin and subjected to MS analysis as described in (Shevchenko *et al.*, 2007). Peptide MS signatures were used to identify the proteins through Mascot Server (Perkins *et al.*, 1999).

(b) Protein gel and Western blot depicting MtbCpn60.1 expression in MGM100 (pMtbCpn1). MtbCpn60.1 expression was confirmed by probing lysates of IPTG-induced cells with the custom synthesized anti-MtbCpn60.1 antibody.

(c) Cellular levels of overexpressed chaperonins. The protein levels of GroEL and MtbCpn60.2 were quantified by relative densitometric analysis of the CBB stained gel. The bars represent the chaperonin band intensity relative to the sum of intensities of all protein bands in the respective lanes.

(d) IPTG-independent *groEL* expression from leaky P_{trc} promoter of pEcoESL. Aliquots of uninduced and induced cells harbouring the pEcoESL plasmid were collected and lysed. Total protein extracted from the cells were resolved on an SDS-polyacrylamide gel to check for

leaky expression of the plasmid-borne *groES* and *groEL* genes in the absence of IPTG induction.

Lanes: M_T – Unstained Protein Molecular Weight Markers (Thermo Scientific, USA), M_N – Blue Prestained Protein Standard, Broad Range (New England BioLabs, UK), V – MGM100(pTrc99a), E – MGM100(pEcoESL), C1 – MGM100(pMtbCpn60.1), C2 – MGM100(pMtbCpn60.2), U – Total protein from uninduced cells, I – Total protein from induced cells.

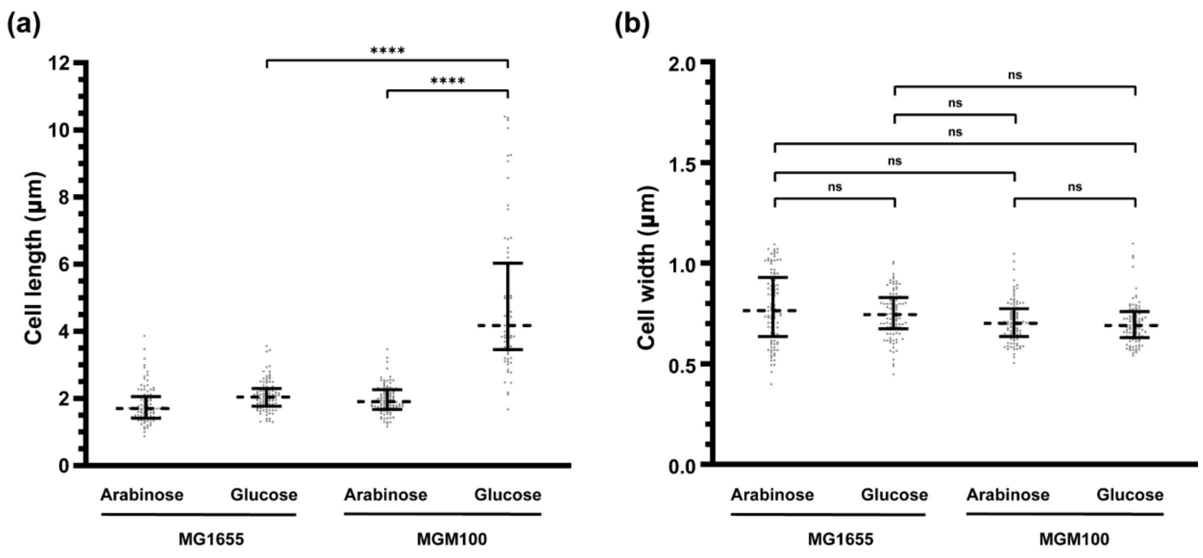

Figure S3: Effect of GroEL depletion on *E. coli* cell morphology and size.

Overnight cultures of the *E. coli* strains MG1655 and MGM100 were sub-cultured for 5 h in LB-arabinose and LB-glucose-DAP. Cultures were diluted 100-fold, and examined by phase contrast microscopy at 1000X for cell morphology and size. Cell lengths and widths were measured for 100 cells using the line measurement tool in Fiji image processing software (Schindelin *et al.*, 2012), and subjected to statistical analyses using GraphPad Prism (www.graphpad.com). Statistical significance at $P < 0.05$. **** $P < 0.0001$, ns $P > 0.05$.

(a) Scatterplots showing distribution of cell lengths, median cell lengths (----) and inter-quartile range (error bars).

(b) Scatterplots showing distribution of cell widths, median cell widths (----) and inter-quartile range (error bars).

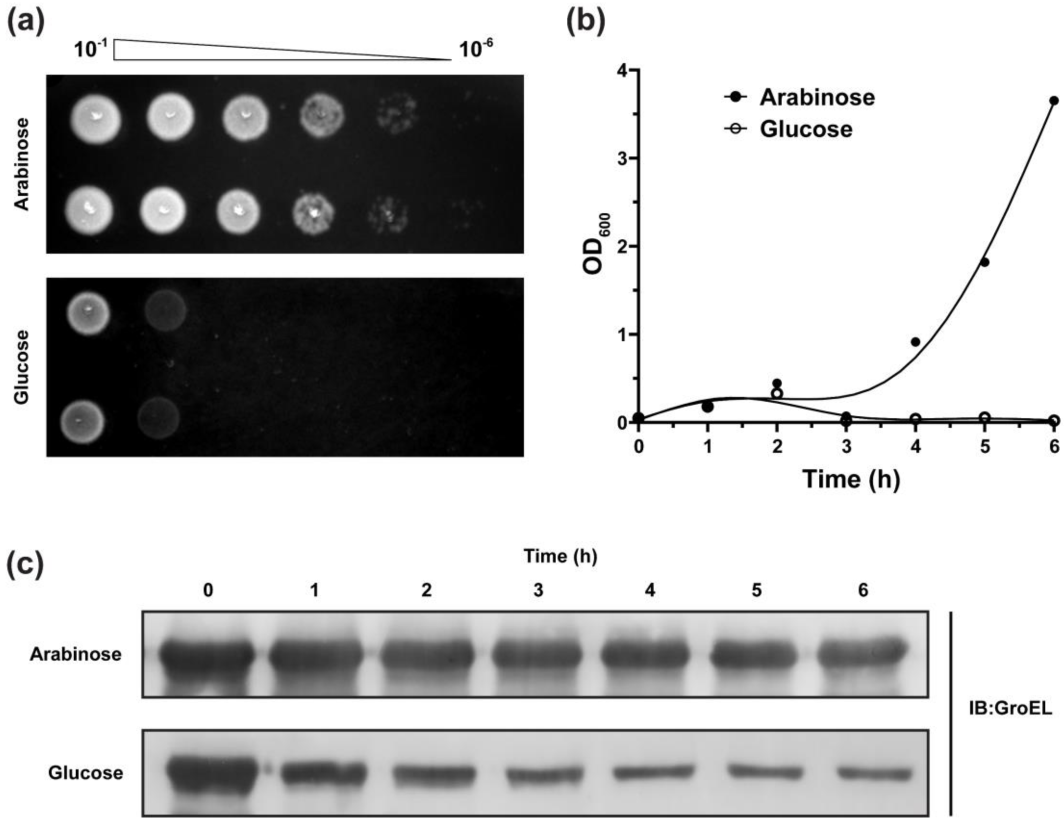

Figure S4: Effect of GroEL depletion on *E. coli* growth and proliferation.

(a) MGM100 overnight cultures were normalised for absorbance (OD_{600}) and subjected to 10-fold serial dilutions (10^{-1} to 10^{-6}). The dilutions were spotted onto LB-agar plates supplemented with 0.2% L-arabinose or 0.2% D-glucose, the plates incubated at 37 °C and analysed for colony formation.

(b) Normalised overnight cultures of *E. coli* MGM100 were sub-cultured into either LB-arabinose or LB-glucose medium to an initial absorbance of 0.05 OD units, and culture growth was monitored at 37 °C by OD_{600}measurement at 1 h intervals.

(c) Total protein extracts were prepared from culture samples collected at intervals of 1 h, resolved on a 10% SDS-polyacrylamide gel followed by immunoblotting (IB) with anti-GroEL antibody.

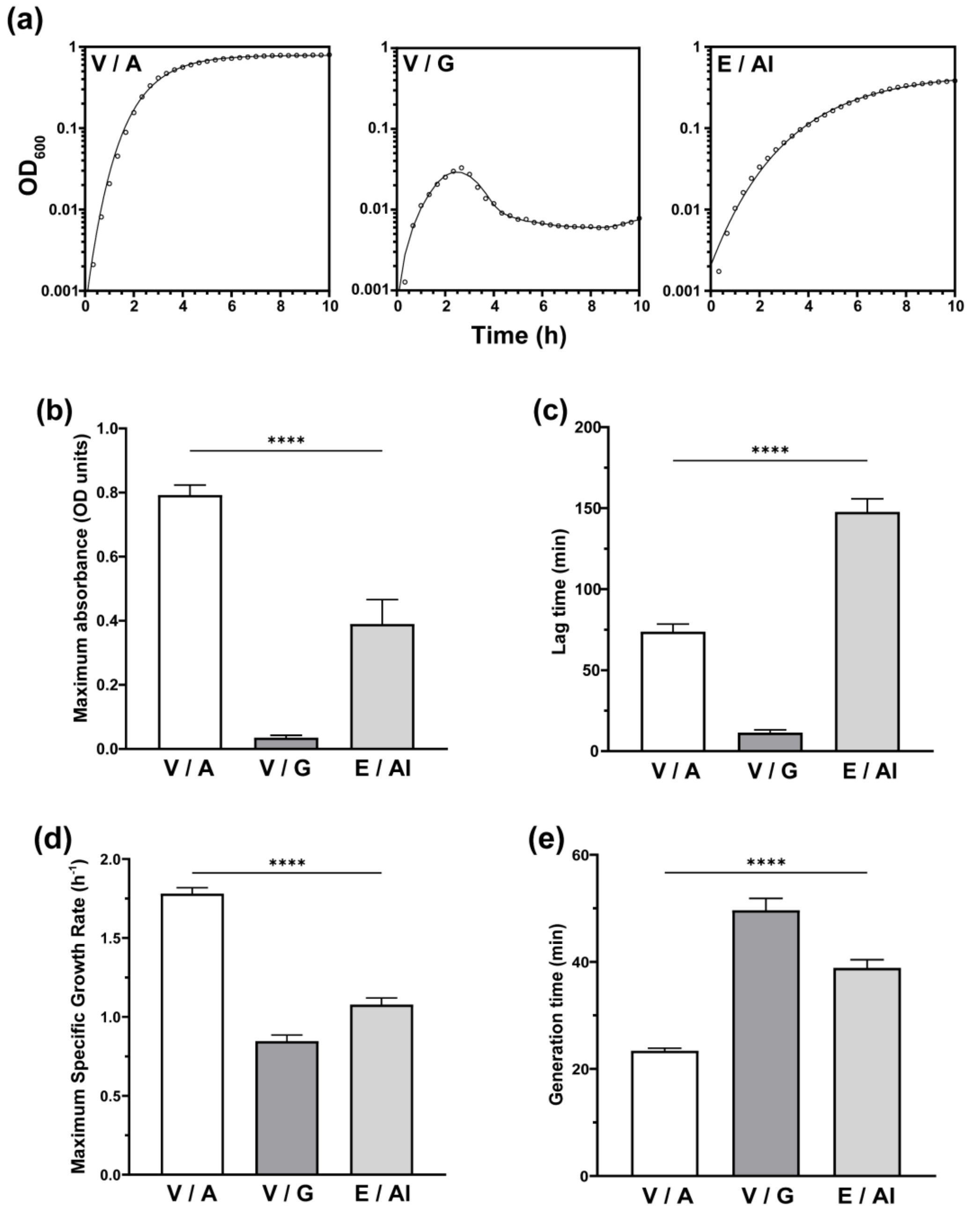

Figure S5: Effect of cellular GroEL levels on *E. coli* growth and proliferation.

Overnight culture of the *groES-groEL* overexpressing strain (E / AI) was subcultured in arabinose-supplemented medium, and grown in parallel with the GroEL-normal (V / A) and GroEL-depleted (V / G) cultures of the *E. coli* MGM100 (pTrc99a) strain. The cultures were induced with 100 µM IPTG for 10 h and OD_{600} measured every 20 minutes.

(a) Semi-log plots illustrating growth curves of strains expressing *groES-groEL* at different levels. Data points represent mean OD600 values of three biological replicates plotted on a logarithmic y-axis and curves represent best fit of the Gompertz growth model.

(b) – (e) Comparison of growth parameters of strains expressing *groES-groEL* at different levels. The Gompertz equation was used to compute growth parameters illustrated as bar

graphs. Bars represent mean ± SEM of three biological replicates. **(b)** Maximum population depicts maximum OD_{600} for each of the cultures. **(c)** Lag time depicts the duration of lag phase before the cultures progressed to exponential growth. **(d)** Maximum specific growth rate depicts the highest specific growth rate achieved during exponential growth. **(e)** Generation time depicts the average doubling time observed during exponential growth.

Data: Statistical significance determined at $P < 0.05$ (Student's t test). **** $P < 0.0001$.

Lanes: V / A – MGM100(pTrc99a) in LB-arabinose, V / A – MGM100(pTrc99a) in LB-glucose-IPTG, E / AI – MGM100(pEcoESL) in LB-arabinose-IPTG.

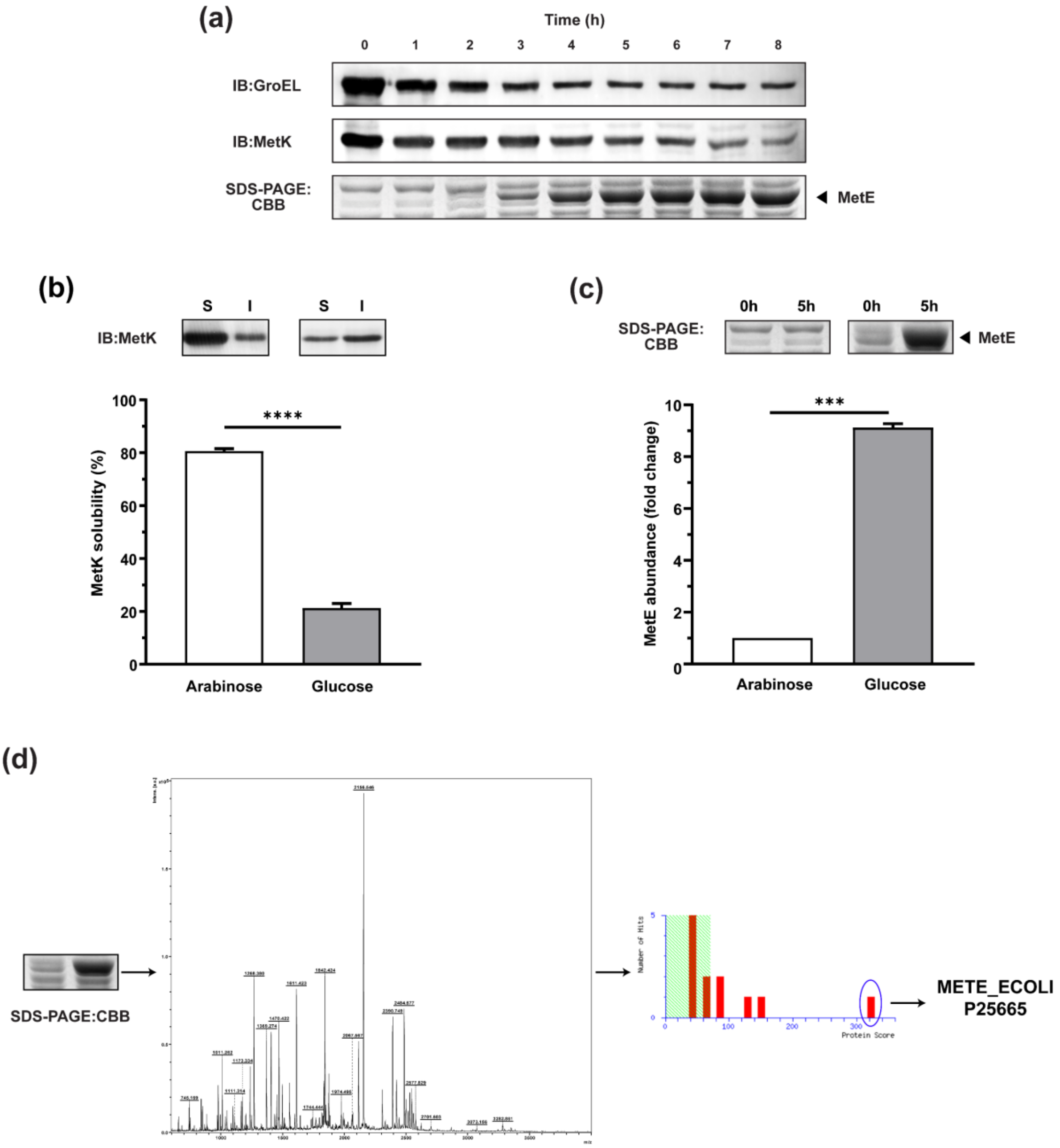

Figure S6: Effect of GroES-GroEL depletion on MetK folding and function.

Parallel cultures of the *E. coli* MGM100 strain were grown in LB-arabinose or LB-glucose-DAP medium (to yield normal or depleted levels of GroEL respectively).

(a) GroEL depletion associated changes in levels of endogenous MetK and MetE. Samples were collected from GroEL-depleted MGM100 culture at intervals of 1 h, followed by extraction of total protein. Total protein extracts were resolved on SDS-polyacrylamide gels, which were subjected to Coomassie Brilliant Blue (CBB) staining for MetE and immunoblotting (IB) for GroEL and MetK.

(b) GroEL-depletion-associated changes in MetK solubility. Samples were collected from both GroEL-normal and GroEL-depleted MGM100 cultures after 5 h of incubation, followed by extraction of total protein, fractionation of the extracts into soluble (S) and insoluble (I) fractions, resolution of the fractions on SDS-polyacrylamide gels and immunoblotting with anti-

MetK antibody. The bar graph shows MetK solubility as estimated by relative densitometric analysis of the immunoblots. Fractional solubility computed as the percentage of MetK present in the soluble fraction.

(c) GroEL-depletion associated changes in MetK function. Samples were collected from both GroEL-normal and GroEL-depleted cultures at 0 h and 5 h of incubation, followed by extraction of total protein, resolution of the extracts on SDS-polyacrylamide gels, and CBB staining of the gels to observe MetE overexpression. MetE abundance at 5 h was quantified by densitometric analysis relative to 0 h. Fold change in MetE abundance in GroEL-depleted MGM100 cells was estimated in comparison to GroEL-normal cells.

(d) MS analysis of the MetE protein band. The MetE protein band was excised from the gel, subjected to in-gel proteolysis, the peptides were then extracted and subjected to MS analysis (Shevchenko *et al.*, 2007). Protein identity was confirmed with the help of Mascot Server (Perkins *et al.*, 1999).

Data: Images and data representative of three independent experiments. The bar graphs present data as Mean ± SEM. Statistical significance determined at $P < 0.05$ (Student's t test). **** $P < 0.0001$, *** $0.0001 < P < 0.001$.

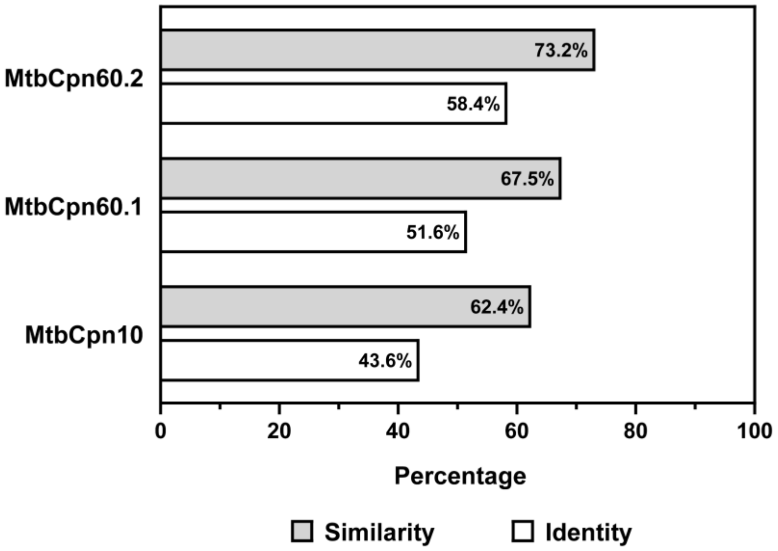

Figure S7: Sequence similarity between *E. coli* and *M. tuberculosis* chaperonin protein homologs.

Bar graph illustrating global sequence similarity (grey) and identity (white) computed from pairwise sequence alignments using the EMBOSS Needle tool (Needleman and Wunsch, 1970) for protein sequences retrieved from the NCBI Protein database (Sayers *et al.*, 2022). Protein [Accession]: GroEL [NP_418567.1], MtbCpn60.1 [NP_217934.1], MtbCpn60.2 [NP_214954.1], GroES [NP_418566.1], MtbCpn10 [NP_217935.1].

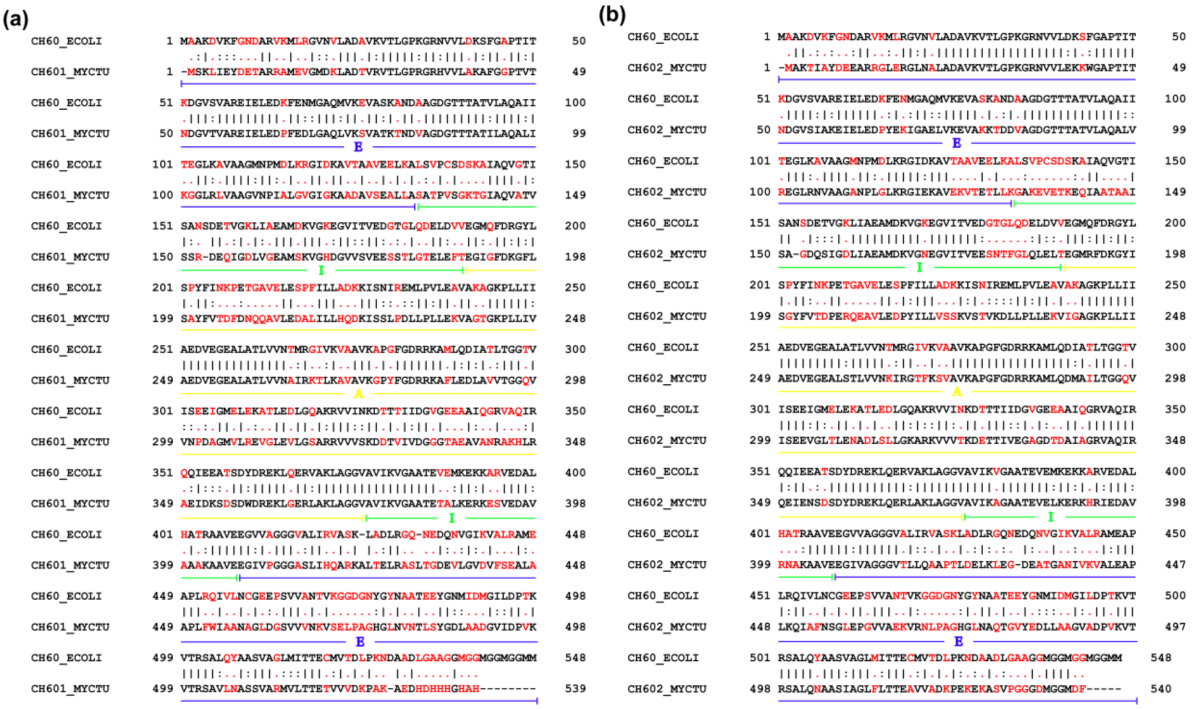

Figure S8: Pairwise alignments of *M. tuberculosis* Cpn60 protein sequences with GroEL.

Protein sequences retrieved from the NCBI Protein database (Sayers *et al.*, 2022) were aligned using the EMBOSS Needle tool (Needleman and Wunsch, 1970). Similar residues are in black while residue variations are in red. The sequence stretches corresponding to the three domains are marked with A (yellow) for apical, I (green) for intermediate and E (blue) for equatorial. Protein [Accession]: GroEL [NP_418567.1], MtbCpn60.1 [NP_217934.1], MtbCpn60.2 [NP_214954.1].

(a) Pairwise alignment of MtbCpn60.1 (CH601_MYCTU) with GroEL (CH60_ECOLI).

(b) Pairwise alignment of MtbCpn60.2 (CH602_MYCTU) with GroEL (CH60_ECOLI).

Supplementary Tables

Table S1: Growth parameters computed for liquid cultures of chaperonin expression strains.

Strain \ Growth parameter		**Maximum specific growth rate (h^{-1})**	**Doubling time (min)**	**Maximum population $(OD\ units)$**	**Lag time (min)**
**DAP –**	**V/arabinose**	1.86 ± 0.17	22.4 ± 2.1	0.93 ± 0.01	74.4 ± 3.0
	**V/glucose**	1.06 ± 0.07	39.4 ± 2.6	0.03 ± 0.01	53.3 ± 5.8
	**E**	1.96 ± 0.04	21.2 ± 0.4	1.13 ± 0.03	63.3 ± 5.8
	**C1**	1.49 ± 0.23	28.3 ± 4.8	0.18 ± 0.03	54.2 ± 0.3
	**C2**	1.48 ± 0.09	28.3 ± 1.7	1.27 ± 0.11	106.1 ± 16.5
**DAP +**	**V/arabinose**	1.88 ± 0.13	22.2 ± 1.6	0.92 ± 0.01	73.7 ± 6.4
	**V/glucose**	0.94 ± 0.02	44.4 ± 0.8	0.07 ± 0.03	55.5 ± 7.7
	**E**	1.86 ± 0.07	22.4 ± 0.9	1.18 ± 0.02	70.0 ± 9.9
	**C1**	1.61 ± 0.01	25.8 ± 1.7	0.23 ± 0.06	59.2 ± 10.1
	**C2**	1.56 ± 0.04	26.6 ± 0.6	1.21 ± 0.06	103.3 ± 5.8

Cultures of the chaperonin expression strains (V, E, C1, C2) were grown in LB-glucose-IPTG, in parallel with the GroEL-normal culture (V/arabinose). The cultures were induced with 100 μM IPTG and incubated at 37 °C. Growth curves were obtained by periodic measurement of OD_{600} every 10 mins. The curves were then fitted using the Gompertz model and the Gompertz equations were subsequently used to estimate critical growth parameters for each of the cultures. Data represents mean ± SD of three biological replicates. Strains: V – MGM100(pTrc99a), E – MGM100(pEcoESL), C1 – MGM100(pMtbCpn60.1), C2 – MGM100(pMtbCpn60.2).

Supplementary References

Kumar, C.M.S., Chugh, K., Dutta, A., Mahamkali, V., Bose, T., Mande, S.S., *et al.* (2021) Chaperonin Abundance Enhances Bacterial Fitness, *Frontiers in Molecular Biosciences*, **8**, 669996. https://doi.org/10.3389/fmolb.2021.669996.

McLennan, N. and Masters, M. (1998) GroE is vital for cell-wall synthesis, *Nature*, **392**(6672), 139. https://doi.org/10.1038/32317.

Needleman, S.B. and Wunsch, C.D. (1970) A general method applicable to the search for similarities in the amino acid sequence of two proteins, *Journal of Molecular Biology*, **48**(3), 443–453. https://doi.org/10.1016/0022-2836(70)90057-4.

Perkins, D.N., Pappin, D.J.C., Creasy, D.M. and Cottrell, J.S. (1999) Probability-based protein identification by searching sequence databases using mass spectrometry data, *Electrophoresis*, **20**(18), 3551–3567. https://doi.org/10.1002/(SICI)1522-2683(19991201)20:18<3551::AID-ELPS3551>3.0.CO;2-2.

Sayers, E.W., Bolton, E.E., Brister, J.R., Canese, K., Chan, J., Comeau, D.C., *et al.* (2022) Database resources of the national center for biotechnology information, *Nucleic Acids Research*, **50**(D1), D20–D26. https://doi.org/10.1093/nar/gkab1112.

Schindelin, J., Arganda-Carreras, I., Frise, E., Kaynig, V., Longair, M., Pietzsch, T., *et al.* (2012) Fiji: an open-source platform for biological-image analysis, *Nature Methods*, **9**(7), 676–682. https://doi.org/10.1038/nmeth.2019.

Shevchenko, A., Tomas, H., Havliš, J., Olsen, J. V. and Mann, M. (2007) In-gel digestion for mass spectrometric characterization of proteins and proteomes, *Nature Protocols*, **1**(6), 2856–2860. https://doi.org/10.1038/nprot.2006.468.
